# Lifecycle management of orphan drugs approved in Japan

**DOI:** 10.1186/s13023-022-02456-w

**Published:** 2022-07-29

**Authors:** Kiyoshi Seki, Hiroshi Suzuki, Seiji Abe, Chikako Saotome

**Affiliations:** Kyoto University Graduate School of Medicine, 53 Shogoin Kawahara-cho, Sakyo-ku, Kyoto-shi, Kyoto, 606-8507 Japan

**Keywords:** Orphan drug, Life cycle management, New indication approval, Patent, Re-examination term, Market exclusivity, Generics

## Abstract

**Background:**

The development of orphan drugs (ODs) is challenging from both development and business perspectives because of their small patient populations. To overcome such business challenges, lifecycle management (LCM), which maximizes profits by increasing sales and extending product lifetimes, is important to overcome the business challenges arising from their small patient populations. To clarify the activities of the LCM of ODs, we investigated additional indications that contribute to market expansion and marketing exclusivity using the patent extension and re-examination system of ODs approved in Japan between 2004 and 2019.

**Results:**

The 203 ODs consisting of 173 active ingredients were approved in Japan between 2004 and 2019. Sixty-eight (39%) of the 173 active ingredients have additional indications, of which 57 have at least one non-OD indication. Three-fourths of the 203 ODs had patent rights, and most of them included substance or use claims. Although the re-examination period for most ODs was 10 years after the approval, most patents had a longer duration than the re-examination period.

**Conclusions:**

Pharmaceutical companies were actively adding non-OD indications and were emphasizing the use of patent rights by registering extensions of substance or use patents for exclusive marketing periods. These results indicate that LCM through the addition of indications and registration of patent extensions is carried out as a strategy for many ODs in Japan, similar to the LCM of general non-ODs.

## Background

Rare diseases are currently estimated at more than 7000, and 95% of them have no appropriate treatment available [1, 2]. The development of orphan drugs (ODs) is therefore strongly demanded. However, the development of ODs is challenging from both development and business perspectives because of their small patient populations. In Japan, the OD designation program was established in 1993 under the “Act on Securing Quality, Efficacy and Safety of Products including Pharmaceuticals and Medical Devices” (also known as the Pharmaceutical and Medical Devices Law, hereinafter referred to as the Pharmaceutical Affairs Law). The OD designation provides pharmaceutical companies with incentives such as priority review, subsidies, and a re-examination period (i.e., market exclusivity) of up to 10 years, which is 2 years longer than the period for usual new molecular entities.

The efficiency of drug development has recently been declining [3]. Therefore, lifecycle management (LCM), which is a marketing method for maximizing profits through increasing sales and extending product life, has become increasingly important for pharmaceuticals. Specifically, LCM in pharmaceuticals includes the expansion of indications, the addition of dosage forms, and the improvement of dosage and administration after the approval of a new drug to maximize sales and expand the product’s lifespan. In the development of ODs, reducing the cost by adding new indications to existing drugs or expanding the number of patients eligible for the new indications as LCM is very important with respect to the profitability of development investment. In particular, the additional approval of non-OD indications is expected to increase sales dramatically by expanding the number of target patients. We previously reported a case study in which the first ODs approved for rare diseases such as Crohn’s disease and Castleman’s disease were subsequently approved for rheumatoid arthritis, which is a common disease, resulting in increased sales as an effect of LCM [4].

With respect to exclusivity rights that contribute to the extension of product life, there is the re-examination period corresponding to data protection in the United States under the Pharmaceutical Affairs Law. As an incentive for OD development, pharmaceutical companies feel the most benefit from a 2-year extension of the re-examination period [5]. And the effect of the 7-year OD market exclusivity provision in the United States has also been reported to be relatively modest [6]. In addition, patents play a major role in ensuring the exclusivity of drugs. The scope of the patent rights is described in the claims, and a single patent usually includes multiple claims. The validity of a patent varies depending on the type of invention described in each claim. In terms of pharmaceuticals, substance patents, which protect the active ingredients of the drug itself, and use patents, which protect the indication of the drug, are important. In the case of Japanese patents, use patents are described as pharmaceutical composition (use) claims. The patent rights expire 20 years from the filing date; however, patents on drugs cannot be enforced until the drugs have been approved for manufacture and sale by authorities such as the Pharmaceuticals and Medical Devices Agency (PMDA) in Japan. To recover this period, patent law allows the duration of patent rights to be extended for up to 5 years in the case of pharmaceuticals. The patent extension system differs from country to country. In the United States, which is the largest market for pharmaceuticals, only one patent can be extended for one pharmaceutical product at the time of the first pharmaceutical approval of such product. By contrast, in Japan, the number of patents that can be extended and the number of times the term can be extended for patents that meet the requirements for registration of extension are unlimited. The main requirements for registration of a patent extension in Japan are that (1) the term of the patent right has not yet expired, (2) there is a period during which the patented invention cannot be used, and (3) the patent is for a pharmaceutical or regenerative medicine product. In the case of ODs, numerous cases are expected in which utilization of the patent extension system is difficult because of the short period of the clinical trial and application for approval under priority review, etc. Consequently, the patent extension system might be used when the patent extension period exceeds the re-examination period [7].

Although the use of LCM is important in the development of ODs, a complete picture of LCM of ODs has not yet been reported in terms of additional indications or marketing exclusivity when the patent extension system and re-examination period are considered. Therefore, we aim to clarify the OD LCM activity in Japan through investigations of additional indications and marketing exclusivity using the patent extension and re-examination system.

## Methods

### Approved ODs

Using the list of designated ODs [8] prepared by the National Institutes of Biomedical Innovation, Health and Nutrition on September 17, 2019, we selected 203 ODs approved for marketing and manufacturing among 276 OD candidates designated by the Ministry of Health, Labour and Welfare of Japan after April 1, 2004, when the PMDA was established. We then identified the active ingredients of the 203 ODs using the list of approved new drugs [9] prepared by the PMDA.

### Additional indications

For each active ingredient, the PMDA’s list of new drug approvals [9] and the interview forms were used to investigate whether an additional indication was added, the distinction between OD and non-OD indications, the order in which the indications were obtained, the number of additional approvals, the pharmaceutical application category, and the target disease area for which the indication was approved.

### Market exclusivity

We searched patents for each of the 203 ODs using J-PlatPat (Industrial Property Information and Training Center), JP-NET Web (Japan Patent Data Service), Orange Book (United States Food and Drug Administration), and Cortellis (Clarivate Analytics) and then investigated the existence of the patent related to each OD, the registrations of patent extensions, and the protection term. Next, we classified patent claims into eight categories: pharmaceutical substances, pharmaceutical compositions (uses), pharmaceutical compositions (general preparations), pharmaceutical compositions (specific preparations), other substances, manufacturing methods (pharmaceuticals), manufacturing methods (intermediates, etc.), and methods. All authors conducted the claim classification investigation independently and in duplicate, and any differences in opinion were decided by the consensus of all authors.

The re-examination period of each OD was investigated using the minutes of the Pharmaceutical Affairs and Food Sanitation Council [10] and the examination records for each drug. Then, we compared the patent term and re-examination term to determine which term was longer.

### Generics

We used Nikkei Medical’s Prescription Drug Dictionary [11] to investigate whether any generics were available on the market with the same indications and indications for ODs (for which there is no marketing exclusivity) whose re-examination and patent terms (including extension terms) had expired as of the end of January 2021.

## Results

### Approved ODs

The 203 approved ODs consisted of 173 active ingredients. In the following, pharmaceutical approvals for additional indications are granted for each active ingredient; thus, the results of the investigation on additional indications are described using 173 ingredients as the population. In addition, because patent extensions are registered on a product-by-product basis, the results of the investigation on patents are based on a population of 203 OD products.

### Additional indications

Of the 173 ingredients, 68 (39%) had additional indications (Table 1). However, 105 (61%) had only one OD indication, which was obtained at the time of approval of the new drug, and no other indication.

**Table 1:** Existence and numbers of additional indications

	Ingredient (%)		Times	
**No additional indication**	105 (61)		0.0	
**With additional indication**	68 (39)		2.0	
NonOD → OD	34 (20)	57 (33)	1.7	2.2
OD → nonOD	23 (13)		3.0	
OD → OD	11 (6)		1.1	

For the 68 ingredients that received additional indications, 57 ingredients (33%) had additional approved non-OD indications. The order in which the OD and non-OD indications were obtained for the 57 components was as follows: 34 components (20%) had non-OD indications approved before OD approval, and 23 components (13%) had non-OD indications added after OD approval. The group with earlier non-OD-indication approval included Avastin^®^ (Bevacizumab, non-OD indication: advanced or recurrent colorectal cancer that is not curatively unresectable, in 2007 → OD indication: malignant glioma, 2013, Chugai Pharmaceutical Co., Ltd.) and Humira^®^ (Adalimumab, non-OD indication: rheumatoid arthritis (limited to patients with inadequate response to existing therapies), in 2008 → OD indication: pyogenic sweat gland inflammation, in 2019, Eisai Co., Ltd.). Examples of drugs with a later non-OD-indication approval include Opdivo^®^ (Nivolumab, OD indication: unresectable malignant melanoma, in 2014 → non-OD indication: unresectable advanced or recurrent non–small-cell lung cancer, in 2016, Ono Pharmaceutical Co., Ltd.) and Remicade^®^ (Infliximab, OD indication: Crohn’s disease, in 2002 → non-OD indication: rheumatoid arthritis, in 2003, Mitsubishi Tanabe Pharma Corporation). However, 11 ingredients (6%) had only other OD indications added. Examples include Darzalex^®^ (Daratumumab, OD indication: relapsed or refractory multiple myeloma, in 2017 → OD indication: multiple myeloma, in 2019, Janssen Pharmaceutical K.K.) and Xalkori^®^ (Crizotinib, OD indication: ALK fusion gene-positive advanced non–small-cell lung cancer, in 2012 → OD indication: ROS1 fusion gene-positive unresectable advanced or recurrent non-small-cell lung cancer, in 2017, Pfizer Japan Inc.). The average number of additional approvals per active ingredient was 2.0 for the 68 ingredients with additional indications. The average number of indications was 2.2 for the 57 ingredients with non-OD indications and 1.1 for the 11 ingredients without non-OD indications. The average number of additional approvals for ingredients with non-OD indications was twice as high as that for ingredients without non-OD indications.

Table 2 shows the disease areas of the 173 active ingredients. In four disease areas—specifically, field 5 (urogenital and anal medicines), radiopharmaceuticals, gene therapy, and bio-quality—no OD approval was obtained. However, there were more than 10 approvals for the number of active ingredients in field 2 (drugs for cardiovascular, Parkinson’s disease, and Alzheimer’s disease), field 3–1 (drugs for central nervous system (CNS) and peripheral nervous system (PNS)), field 6–1 (drugs for respiratory, allergy, and sensory organs with inflammatory diseases), field 6–2 (hormones, and drugs for metabolic diseases), anticancer, and AIDS drugs. These six areas accounted for a total of 144 ingredients (83%), with the anticancer group accounting for a particularly high 66 ingredients (38%).

**Table 2:** Characteristics of additional indications in each disease area

Categories	1	2	3–1	3–2	4	5	6–1	6–2	Imaging agent	Radiophar-maceutical	Anticancer	Anti-HIV	Vaccine	Blood product	Gene therapy	Bio-quality	Total
Total	6	15	17	2	6	0	13	19	1	0	66	14	9	5	0	0	173
**No additional indication**	1	8	10	1	5	0	4	15	0	0	36	12	9	4	0	0	105
**With additional indication**	5	7	7	1	1	0	9	4	1	0	30	2	0	1	0	0	68
NonOD → OD	3	4	6	0	1	0	5	3	0	0	11	1	0	0	0	0	34
OD → nonOD	2	2	0	1	0	0	2	1	0	0	13	1	0	1	0	0	23
OD → OD	0	1	1	0	0	0	2	0	1	0	6	0	0	0	0	0	11

The PMDA classifies drug categories as follows: 1: gastrointestinal, topical, and immunosuppression 2: cardiovascular, Parkinson's, and Alzheimer's; 3–1: central nervous system and peripheral nervous system (except for anesthetics), 3–2: anesthetic, ophthalmic and otic; 4: antibacterial, anti-parasitic, and anti-viral (except for HIV); 5: urogenital and anal; 6–1: respiratory, anti-allergy, and anti-inflammatory; 6–2: hormones and metabolic disease

Of the diseases with an approved OD, additional indications were obtained for all diseases except vaccines. The areas where the percentage of additional indications was higher than that of no additional indications were field 1 (gastrointestinal, topical, and immunosuppression medicines, 83%), field 6–1 (drugs for respiratory, allergy, and sensory organs with inflammatory diseases, 69%), and field 3–2 (anesthetic, ophthalmic and otic medicines, 50%). In the field of anticancer, which had the highest number of OD approvals, 30 drugs (45%) had additional indications, of which 13 (20%) had non-OD indications added after OD approval.

### Market exclusivity

As a result of the search for relevant patents of the 203 ODs, we found that 154 (76%) ODs had patents and 49 (24%) ODs had no patents. Of the 154 ODs with patents, 125 had a patent extension registered and 29 did not. The average numbers of patents were 2.1 for the 154 ODs, 2.2 for the 125 ODs with an extension, and 1.4 for the 29 ODs without an extension. The 21 of 29 ODs without a patent extension could not be registered because their patents had not been granted at the time of regulatory approval.

Next, we classified the claims of each patent right and investigated what kinds of inventions were protected (Table 3). We found that 108 items (70%) were protected by the invention of the pharmaceutical composition (use), which protects the use of medicine to treat a certain disease. In addition, the number of ODs protected by the invention of pharmaceutical substances was 92 (60%). The majority of ODs protected by patented inventions of either pharmaceutical substances or pharmaceutical compositions (uses) was 139 (90%) of 154 items (not shown in Table 3). The results for ODs with a patent extension showed a similar tendency: 91 ODs (73%) by pharmaceutical composition (use), 84 ODs (67%) by pharmaceutical substances, 118 ODs (94%) by pharmaceutical substances or pharmaceutical compositions (uses).

**Table 3:** Summary of the characteristics of claims in patents

Category	Total		With extension		No extension	
	(*N* = 154)	(%)	(*N* = 125)	(%)	(*N* = 29)	(%)
Pharmaceutical substances	92	60	84	67	8	28
Pharmaceutical compositions (uses)	108	70	91	73	17	59
Pharmaceutical compositions (general preparations)	63	41	58	46	5	17
Pharmaceutical compositions (specific preparations)	43	28	29	23	14	48
Other substances	46	30	39	31	7	24
Manufacturing methods (pharmaceuticals)	75	49	59	47	16	55
Manufacturing methods (intermediates, etc.)	13	8	12	10	1	3
Methods	14	9	13	10	1	3

Among the 29 ODs protected by patents without extension, the largest number of ODs was 17 (59%) protected by inventions of pharmaceutical compositions (uses), followed by 16 (55%) protected by inventions manufacturing methods (pharmaceuticals). By contrast, only 8 ODs (28%) were protected by inventions of pharmaceutical substances.

The re-examination period for most of the ODs was 10 years after the approval; there were four exceptions. These four ODs were Prograf^®^ (Tacrolimus), Velcade^®^ (Bortezomib), Lynparza^®^ (Olaparib), and Darzalex^®^ (Daratumumab, genetical recombination), and their re-examination periods ranged from four to 8 years. In each case, another indication for the same active ingredient was approved prior to this approval and the remaining period was the re-examination period.

To determine whether the patent right or the re-examination period is longer in terms of the exclusivity period, we compared the expiration date of the patents with the end date of the re-examination period. As shown in Table 4, 103 (82%) of 125 ODs with registered patent extensions and 23 (79%) of 29 ODs without registered patent extensions had patent terms that continued beyond the end of the re-examination period. The average patent term was 2.9 years longer than the re-examination term, with the shortest term being 16 days and the longest being 12.8 years.

**Table 4:** Comparison of exclusive terms between patents and re-examinations

	Re-examination > patents	Re-examination < patents	Total
Total	28	126	154
With extension	22	103	125
No extension	6	23	29

We here describe the results of a comparison of the patent terms and re-examination terms for 105 ODs that have no additional indication and for which LCM by exclusivity is more important. Of the 105 ODs, 79 (75%) were patented, of which 63 (60%) products had patent terms that continued beyond the end of the re-examination period. Sixty-two ODs (59%) were registered for patent extension, of which 48 (46%) products had longer patent terms than the re-examination period. However, 26 ODs (25%) had exclusivity terms only by re-examination period. Regarding disease fields, seven were in field 6–2 (hormones, and drugs for metabolic diseases) and four each were in field 2 (drugs for cardiovascular, Parkinson’s diseases, and Alzheimer’s disease), field 3–1(drugs for CNS and PNS), and the anticancer field. The ODs in field 6–2 (hormones, and drugs for metabolic diseases) were only ultra-orphan drugs whose number of patients was less than 200. In addition, many of the drugs in field 6–2 (hormones, and drugs for metabolic diseases), the anticancer field, and field 3–1 (drugs for CNS and PNS) (e.g., Vidaza^®^ (Azacitidine) and Diacomit^®^ (Stiripentol)) were included in the list of drugs for which development companies were solicited or requested to develop on the basis of the results of a review by the Review Committee on Unapproved and Off-label Drugs with High Medical Needs [12].

In addition, by integrating the results of the investigations of the additional indications and the patent rights, we found that 177 ODs (87% of 203 ODs) were the subject of LCM using either additional indications or a patent extension.

### Generics

Of the 203 ODs, 39 were outside the re-examination period as of the end of January 2021. Of these 39 ODs, 25 had expired patent periods. Because two of them had been canceled for approval due to discontinuation of the manufacture and sales, 23 of the ODs were not protected by the re-examination period or their patent term. Of these 23 ODs, only three ODs had generics on the market. Specifically, Actonel^®^ (Risedronic acid) was indicated for the treatment of Paget’s disease of bone (non-OD indication: osteoporosis), Prograf^®^ (Tacrolimus) was indicated for the treatment of myasthenia gravis and spring catarrh with inadequate response to antiallergic drugs (non-OD indication: inhibition of rejection of kidney, liver, and other transplants, rheumatoid arthritis, and others), and Remicade^®^ (Infliximab, genetic recombination) was indicated for the treatment of ankylosing spondylitis (non-OD indication: rheumatoid arthritis and many others).

## Discussion

### LCM by adding indications

The results of the investigation of LCM with additional indications showed that the number of active ingredients with multiple indications for ODs was 39% (68 ingredients), which was less than one-half of the total but accounted for a certain number. Of these, the majority (57 ingredients) had non-OD indications with a large number of patients. In addition, disease areas where LCM for additional indications were being conducted included field 2 (drugs for cardiovascular, Parkinson’s disease, and Alzheimer’s disease), field 6–1 (drugs for respiratory, allergy, sensory organs with inflammatory diseases), and the anticancer field.

Among ODs we investigated, 7 products (3 ODs in oncology, 3 ODs in immunology, and 1 OD in others) were included in the top 20 domestic pharmaceutical products [13] in terms of sales in FY2019: Keytruda^®^ (Pembrolizumab), Avastin^®^ (Bevacizumab), Opdivo^®^ (Nivolumab), Samsca^®^ (Tolvaptan), Remicade^®^ (Infliximab), Humira^®^ (Adalimumab), and Prograf^®^ (Tacrolimus). All of these products have non-OD indications, and the disease areas are also the areas with many additional indications in our results. On the basis of these drugs, we discuss how the LCM of additional indications can overcome the sales issue of the OD business.

The anticancer field, which has the largest number of approved ODs, is the largest market, accounting for more than 10% of the domestic market [13]. Anticancer drugs are often expanded by changing the target organs to other indications. In addition, to obtain OD designation, diseases are easily sub-grouped to limit the number of target patients at the genetic level. Furthermore, patients for whom existing therapies are not effective can be collected. Some ODs have been first approved for diseases with prefixes such as “relapsed,” “refractory,” or “gene-positive” in the target disease name (e.g., “relapsed or refractory multiple myeloma” and “ROS1 fusion gene-positive unresectable advanced or relapsed non-small-cell lung cancer”). Because of the ease of these additional indications, ca. half of ODs (30 of 66) in the anticancer field could be obtained additional indications, and the percentage is the highest. That is, anticancer drugs are considered to be the most likely to benefit from LCM utilizing the Japanese OD system. In addition, 11 of the 30 anticancer ODs have been approved by OD indication after approval of non-OD indication. This may imply that after clearing the problem of profitability, the exclusive period would be extended by the additional OD approval. On the other hand, Opdivo^®^ (Nivolumab) obtained approval as an OD for the target disease and subsequently obtained non-OD approval. Opdivo^®^ (Nivolumab) was approved in 2014 for the indication of OD in malignant melanoma, with an estimated 2000 new patients per year. The initial National Health Insurance price was set high at JPY 730,000 per 100 mg because of its novel mechanism of action and high response rate and efficacy in clinical trials and because it was the first drug of clinical significance for malignant melanoma since the mid-1980s [14]. In 2016, Ono Pharmaceutical Co., Ltd. received additional approval for non-small-cell lung cancer (estimated 100,000 new patients per year) in a non-OD indication, expanding the number of patients eligible for the drug and increasing sales to close to JPY 100 billion, making it the fourth-largest-selling medication [15].

In field 6–1 (drugs for respiratory, allergy, and sensory organs with inflammatory diseases), numerous ODs have been approved for diseases with prefixes such as “intractable” in the name of the target disease, such as the acute stage of Kawasaki disease and intestinal Behcet’s disease, for which existing treatments are insufficiently effective. For example, Remicade^®^ (Infliximab) and Humira^®^ (Adalimumab) added rheumatoid arthritis, which is a non-OD indication, and achieved annual sales of JPY 50 billion [15].

In this study, we found that 23 ingredients were approved for non-OD indications after OD indications were approved. The use of development incentives such as priority review and high drug prices (additional payment for new drug creation, etc.) were expected when OD indications were developed. In addition, the number of patients eligible for non-OD indications could be expanded through non-OD development. These findings suggest that the addition of non-OD indications will contribute to increased sales. However, with respect to the order in which indications were acquired, non-OD indications were acquired first in a large number of cases. Specifically, 34 ODs were developed by adding OD indications from non-OD indications. Using the approved non-OD for OD development enabled the problem of profitability to be overcome while meeting the unmet needs of rare diseases and opening new markets by expanding the indication to the OD indication. Bagley et al. investigated additional indications of ODs approved by the Food and Drug Administration (FDA) and reported that the percentage of ODs with additional non-OD indication was 23%, of which the most common order of additional indications for ODs was the first non-OD approval, at 14% [16]. Based on the result of the study, they noted that in some cases, obtaining approvals for additional OD indications to existing non-ODs increased sales of the products through the higher price strategy.

Because the profitability of ODs is a concern, we speculated that the development of ODs with additional indications would be widely used to increase the number of patients. However, the number of ODs with additional indications was 68 of 173, which is less than one-half. Specifically, the percentage of additional indications was low, ranging from 0 to 21%, in areas such as field 4 (antibacterial agents, etc., except for AIDS), field 6–2 (hormonal agents, drugs for metabolic diseases), AIDS medications, vaccines, and blood products. These results can be explained by additional indications for antimicrobials and vaccines being difficult to obtain because of their specificity to their target viruses. Because many ODs in field 6–2 (hormones, and drugs for metabolic diseases) are designed to replenish specific enzymes that have been inactivated, which is the cause of the disease, applying these drugs to other diseases is difficult. These results suggest that there are a certain number of active ingredients for which obtaining additional indications for the LCM of ODs is difficult, depending on the disease field, and that there is a strong need to obtain an exclusivity period.

In summary, the characteristics of LCM by additional indications are as follows: (1) non-OD indications are commonly added, (2) there are many additional indications in fields such as oncology and immunology, which may overcome the problem of sales, and (3) more than one-half of ODs have no additional indications, suggesting a greater need to obtain exclusivity.

### LCM by marketing exclusivity

The results of this study on marketing exclusivity in ODs show that (1) 76% of the ODs have patents, and most of them have been registered for patent extension; (2) many of the ODs are protected by extended patents of pharmaceutical substances and compositions (uses); (3) almost all of the ODs have the longest re-examination period of 10 years; (4) many of the ODs have patent exclusivity periods longer than their re-examination periods; (5) 177 ODs (87%) of all approved ODs have additional indications or patent extensions; and (6) generic ODs are not currently being actively developed in Japan.

In the United States, the registration of patent extensions is restricted; a single patent can be extended only once. By contrast, in Japan, multiple patents can be extended multiple times. Therefore, as the first characteristic, we found that 125 ODs of 154 products with patents were registered for patent extension using the unique Japanese system and that an average of 2.2 patents per product were extended. Asada et al. have reported that the most important incentive for pharmaceutical companies to develop ODs is the longer re-examination period, and they speculated that many ODs were developed under only the exclusive right of the re-examination period [5]. However, our results reveal that three-fourths of the approved ODs were protected by patents and their extensions. Therefore, as with general non-ODs, many ODs obtain marketing exclusivity through LCM using patents and their extensions.

On the other hand, 49 ODs (24% of the 203 approved ODs) were not protected by patents, suggesting that the re-examination period also plays some role.

We here discuss the types of patented inventions. In general, during drug development, a patent application for a pharmaceutical substance is first filed to protect the active ingredient itself. A patent application is then filed for a pharmaceutical composition (use) as an additional indication when a different indication than the original is found. In addition, a patent application is filed for a pharmaceutical composition (specific formulation) such as an extended-release formulation. Because of the small number of target patients and the sufficiently long re-examination period for ODs, we thought that the patent strategy for ODs was not as important as non-ODs. However, our results showed that 90% of the 154 ODs having patents (94% of the products for which extensions were registered) were protected by pharmaceutical substance or use inventions, which are most important for protecting the drug. In addition, the results for additional indications showed that OD approval was the first approval for all items except 34 ODs with prior non-OD approval, suggesting that patent protection was planned from the beginning of drug development and that patents were also emphasized in OD LCM.

The classification of inventions in the patents without extension registration was characterized by the fact that the ratio of manufacturing methods (pharmaceuticals) was as high as 55%, which was second to that of pharmaceutical compositions (uses) at 59%. Many of these ODs were approved by the PMDA before the patent was granted. Thus, we inferred that, even in the late stage of clinical trials, patent applications for use or formulation were actively filed and, as a result, the patent rights were not granted in time for pharmaceutical approval.

Our results that the re-examination period being 10 years in almost all cases revealed that the re-examination period as an OD development incentive seems utilized to the maximum extent. However, the patent duration was longer for 126 ODs. Although the re-examination period is at the longest 10 years from the pharmaceutical approval of the designated OD, the patent extension period is determined by the clinical trial period. Because estimating the clinical trial period accurately in advance is difficult, it is unclear how much of the patent period, including extensions, will remain after regulatory approval. Although a discrepancy may exist between the pharmaceutical companies’ initial intention and the results, at least 126 of 203 products had a longer patent term than the re-examination period as a result. This finding suggests that pharmaceutical companies are emphasizing patent protection as part of LCM rather than relying on the re-examination period in OD development. In addition, small-molecule ODs in the United States are also granted a 7-year market exclusivity period after approval; however, the percentage of products with a longer market exclusivity period than the patent protection period has been declining in recent years [17]. These findings indicate that, in both Japan and the United States, pharmaceutical companies place greater importance on extending the period of exclusivity through protection by the patent term than on re-examination or the market exclusivity period as an incentive to promote OD development. In addition, the effect of excluding generics of the relevant OD is high because most of the inventions are pharmaceutical substance or use patents.

### Summary of LCM by adding indications and registering patent extensions

Regarding limitations of LCM by adding indications and registering patent extensions, this study showed that 26 ODs (13% of the total) have neither additional indications nor patent extensions registered. These 26 products include many drugs in field 6–2 (hormones and drugs for metabolic diseases), where adding other indications might be difficult because of the mechanism of the drugs and where filing patent applications might be difficult from the viewpoint of novelty or non-obviousness of the active ingredients. Therefore, it is important to ensure the exclusivity period for the development of such ODs through the re-examination period.

However, 177 ODs (87% of the 203 ODs), excluding the aforementioned 26 ODs, have an additional indication or patent extension. Thus, the developers of many ODs in Japan are proactively trying to maximize profits not only through development incentives such as the extension of the re-examination period but also through LCM by adding indications or a patent extension.

It should be noted that the regulations regarding OD such as designation, approval and exclusivity in Japan are not exactly the same as those in other countries, thus there may be other LCM strategies in the other region such as the United States and Europe. Regarding non-ODs approved in Japan, it is possible to receive orphan designations in other regions. However, unlike in Japan and in the United States, in Europe, it is necessary to obtain a separate marketing authorization and change the brand name in order to obtain additional indications of rare diseases for existing non-ODs. There are also different regulations in Europe, such as the extension of market exclusivity by obtaining an orphan designation cannot be used in combination with the extension of Supplementary Protection Certificates based on pediatric clinical trials. In this way, regulations or the patent system of each region can affect the LCM strategy of each company.

### OD generics

The re-examination period and the protection by patents and their extensions are used to prevent a decline in sales and profitability resulting from a decrease in market share when a generic is launched. We, therefore, investigated the status of generic drug launches of ODs in Japan. As of the end of January 2021, there were 23 ODs whose re-examination period and patent protection period have expired; among them, only three generics (13%) have been launched. That is, generics of ODs in Japan are not being actively developed. Bagley et al. surveyed FDA-approved ODs, including biologics, for generics and reported that 27.3% of ODs have generics [16]. They found that ODs with generics had twice the number of patients in peak year than ODs without generics, suggesting that the number of patients affects the entry of generics. Sarpatwari et al. pointed out that, for small-molecule ODs in the United States, the launch of generics relative to brand ODs as of 2017 was ~ 50%, which is less than the percentage of generics among non-ODs in the United States [17]. The results of these two studies in the United States suggest that generic entry rates are higher for small molecule drugs and that drug modality also has an impact. Kerr et al. investigated generic entry for small molecule drugs approved by the FDA and found that only ODs with both OD and non-OD indications lower the hazard of generic entry in comparison with non-ODs [18]. The percentage of additional non-OD indications is higher ODs in Japan (33%) than in Bagley's results (23%) [16], which may be due to slower generic entry. Although a direct comparison between the United States and Japan is difficult because of the differences in insurance and drug pricing systems, the development of generic OD products in Japan thus far appears to be less active than that of generic ODs in the United States; thus, pharmaceutical companies can sell their original drugs exclusively in Japan even after the expiration of the exclusive period. However, in Japan, where the population is aging and medical costs continue to increase, the use of generics is being promoted to reduce the impact on public finances; consequently, the use of generics has now reached 79.3% on a volume basis [19]. Therefore, the importance of patent-based LCM will increase for the original OD development companies in the future, when generics are more actively developed in the OD market in Japan.

#### Limitations

There are three main limitations to this study. Concerning the analysis of additional indications, sales and profits of drugs by indication should be investigated to quantitatively evaluate the effectiveness of LCM by additional indications on sales and profitability. However, these data are not available to the public and are difficult to obtain. Therefore, this study only discusses the indirect impact on sales by estimating that the number of patients will increase as a result of the additional indication. Another limitation regarding additional indications is that the 105 drugs, which have only a single indication, include 33 drugs that are approved in the last 3 years of our review (September 2019 – September 2016) and may get approvals for additional indications after our review.

With regard to patent searches, because the so-called patent linkage between new drugs and their related patents is not disclosed in Japan, the comprehensiveness of patent searches for 78 items other than those that are identifiable by pharmaceutical approval and have been registered as extensions (125 items) cannot be guaranteed.

## Conclusion

We found that 87% (177 ODs) of the 203 approved ODs investigated had been subjected to LCM using patent extension systems or additional indications, mainly for non-OD indications. The remaining 13% (26 ODs) were in fields where adding indications is difficult, such as field 6–2 (hormones and drugs for metabolic diseases). In addition, the results suggested that companies were actively adding non-OD indications to active ingredients and disease areas where LCM was possible and were emphasizing the use of patent rights by registering extensions of substance or use patents for exclusive marketing periods. These results indicate that LCM through the addition of indications and registration of patent extensions is carried out as a strategy for many ODs in Japan, similar to the LCM of general non-ODs.

## Data Availability

The datasets used and analyzed during the current study are available from the corresponding author on reasonable request.

## Abbreviations

ODs: Orphan drugs
LCM: Lifecycle management
PMDA: Pharmaceuticals and Medical Devices Agency
FDA: Food and Drug Administration
